# Usability of a Hybrid System Combining P300-Based Brain-Computer Interface and Commercial Assistive Technologies to Enhance Communication in People With Multiple Sclerosis

**DOI:** 10.3389/fnhum.2022.868419

**Published:** 2022-05-26

**Authors:** Angela Riccio, Francesca Schettini, Valentina Galiotta, Enrico Giraldi, Maria Grazia Grasso, Febo Cincotti, Donatella Mattia

**Affiliations:** ^1^Neuroelectric Imaging and BCI Lab, Fondazione Santa Lucia (IRCCS), Rome, Italy; ^2^Servizio Ausilioteca per la Riabilitazione Assistita con Tecnologia, Fondazione Santa Lucia (IRCCS), Rome, Italy; ^3^Multiple Sclerosis Unit, Fondazione Santa Lucia (IRCCS), Rome, Italy; ^4^Department of Computer, Control and Management Engineering Antonio Ruberti, Sapienza University of Rome, Rome, Italy

**Keywords:** assistive technologies, brain-computer interface, multiple sclerosis, P300, Grid 3, end-users, user-centered design, usability

## Abstract

Brain-computer interface (BCI) can provide people with motor disabilities with an alternative channel to access assistive technology (AT) software for communication and environmental interaction. Multiple sclerosis (MS) is a chronic disease of the central nervous system that mostly starts in young adulthood and often leads to a long-term disability, possibly exacerbated by the presence of fatigue. Patients with MS have been rarely considered as potential BCI end-users. In this pilot study, we evaluated the usability of a hybrid BCI (h-BCI) system that enables both a P300-based BCI and conventional input devices (i.e., muscular dependent) to access mainstream applications through the widely used AT software for communication “Grid 3.” The evaluation was performed according to the principles of the user-centered design (UCD) with the aim of providing patients with MS with an alternative control channel (i.e., BCI), potentially less sensitive to fatigue. A total of 13 patients with MS were enrolled. In session I, participants were presented with a widely validated P300-based BCI (P3-speller); in session II, they had to operate Grid 3 to access three mainstream applications with (1) an AT conventional input device and (2) the h-BCI. Eight patients completed the protocol. Five out of eight patients with MS were successfully able to access the Grid 3 *via* the BCI, with a mean online accuracy of 83.3% (± 14.6). Effectiveness (online accuracy), satisfaction, and workload were comparable between the conventional AT inputs and the BCI channel in controlling the Grid 3. As expected, the efficiency (time for correct selection) resulted to be significantly lower for the BCI with respect to the AT conventional channels (Z = 0.2, *p* < 0.05). Although cautious due to the limited sample size, these preliminary findings indicated that the BCI control channel did not have a detrimental effect with respect to conventional AT channels on the ability to operate an AT software (Grid 3). Therefore, we inferred that the usability of the two access modalities was comparable. The integration of BCI with commercial AT input devices to access a widely used AT software represents an important step toward the introduction of BCIs into the AT centers’ daily practice.

## Introduction

Multiple sclerosis (MS) is an autoimmune disease characterized by clinical neurological relapses and progressive loss of motor and sensory function that affects approximately 2.8 million people worldwide (MSIF, 2020). The course of MS is highly variable, but the relapsing and/or progressive course of the disease leads to a long-term sensorimotor disability (Oh et al., 2018). The level of disability can be even magnified by the presence of a characteristic symptom occurring in MS, such as fatigue (Tur, 2016). Fatigue is indeed one of the most common symptoms and is present in almost 80% of the patients with MS (Rottoli et al., 2017), and it can be severe in up to 60% of patients (Hadjimichael et al., 2008). The impact on quality of life (QoL) of such MS long-term consequences is considerably high, especially if one considers the relatively young age of the population affected by MS (Compston and Coles, 2008). Motor disability and fatigue in MS may result in substantial impairment in communication and in the access to digital technologies, thus leading to overall social isolation.

Assistive technology (AT) indicates any product that enables people of all ages with activity limitations in their daily life, education, work, or leisure (Andrich et al., 2013). ATs include various input devices (e.g., mouse emulators, eye-trackers, adapted joysticks, and speech recognition) and specific software (e.g., Grid 3, Smartbox Assistive Technology, 2021) to create customized solutions to overcome disability. ATs are selected and customized based on users’ needs and their motor, sensory, and cognitive impairment (disabilities) and are validated according to the user-centered design (UCD; ISO, 2019) that is defined as an iterative process that involves end-users in all the stages of technology design, development, and testing.

ATs in general can support communication and environmental interaction in people with disabilities due to MS; however, since all conventional AT input devices are muscular dependent, their usability may result to be compromised by the presence of muscular fatigue in these patients (Tur, 2016).

The brain-computer interface (BCI) technology has been demonstrated to provide severely (motor) disabled people with an alternative channel to enhance/restore communication and environmental control that is independent from the physiological peripheral pathways (i.e., nerves and muscles) (Nijboer et al., 2008; Sellers et al., 2010; McCane et al., 2014; Riccio et al., 2015; Schettini et al., 2015; Guy et al., 2018; Wolpaw et al., 2018; Medina-Juliá et al., 2020). Most of the current studies on the feasibility and usability of non-invasive BCIs systems for communication have relied on evoked potentials (EPs) and event-related potentials (ERPs) (e.g., N200; Treder and Blankertz, 2010) as control features (Powers et al., 2015; Allison et al., 2020). More recently, the so-called hybrid BCIs (h-BCIs) have been proposed that utilize more than one physiological signal and/or external signals to increase, for instance, the accuracy and/or the information transfer rate (Choi et al., 2017). The role of these h-BCIs appears particularly relevant in the domain of AT as they can be conceived as an additional input to provide multimodal access (BCI and conventional AT input devices) to AT software for communication and environmental control functionalities (Millán et al., 2010; Riccio et al., 2011, 2015, 2016; Zickler et al., 2011; Thompson et al., 2014; Schettini et al., 2015). The incorporation of the BCI as an input channel to commercial AT software becomes also essential to improve BCI modularity (Liberati et al., 2015) and eventually to better adapt it to users’ sensory, cognitive, and motor profiles (Schreuder et al., 2013).

Till present, patients with MS have been rarely considered as potential end-users of BCIs to support communication/interaction. Martinez-Cagigal et al. (2016) evaluated a P300-based BCI to access a web browser to eventually support communication in patients with MS.

In this study, we evaluated the usability of a newly implemented AT system that adds a P300-based BCI technology to commercial AT input devices (h-BCI system) to eventually access a range of computer applications through a commercial comprehensive AT software for communication and interaction, the Grid 3 platform (Smartbox Assistive Technology, 2021), with the aim to exploit the BCI as an additional input to ATs to address the issues of fatigue limiting the everyday use of ATs in MS end-users with different degrees of disability. The h-BCI system was evaluated according to the UCD metrics and, therefore, in terms of effectiveness, efficiency, and satisfaction.

As such, the proposed hybrid combination of a P300-based BCI with different conventional input devices can eventually enable patients with MS to switch between a muscular–based channel (e.g., joystick control, mouse control, and head tracker control) and a P300-based BCI channel, according to the level of fatigue they experience or their preference. The integration of this h-BCI system with the Grid 3 platform can guarantee universal access to every kind of mainstream application running on a PC (i.e., browsing the Internet and WhatsApp).

## Materials and Methods

### Participants and Routine Clinical Assessment

A total of 13 participants with MS, according to revised McDonald criteria (Thompson et al., 2018), were enrolled in the study (patients with MS; mean age ± *SD* = 51.6 ± 12.9; two women; mean time since diagnosis: 253.4 months, range: 70–399 months).

The inclusion criteria were (1) ≥18 years, (2) diagnosis of MS, and (3) functional limitation in at least one aspect of interpersonal communication or environmental interaction.

The exclusion criteria were (1) global cognitive decline, (2) concomitant aphasia or comprehension deficits, (3) visual field deficits, (4) severe concomitant medical conditions (e.g., fever and infections), and (5) periods of disease exacerbation.

All participants (or their legal guardians when necessary) gave their written informed consent for participation in the study. The study was approved by the Local Ethical Committee (CE/PROG.707) of Fondazione Santa Lucia, IRCCS.

Patients with MS were recruited from those admitted to the AT service of Fondazione Santa Lucia, IRCCS (Rome, Italy), because of their limitations in at least one aspect related to communication and/or environmental interaction. According to the clinical standard care, they underwent a neuropsychological assessment (see Table 1) and were administered with the Fatigue Severity Scale (FSS; Krupp et al., 1989) to assess the severity of fatigue symptoms (scores range: 1–7; low fatigue-high fatigue; mean ± SD = 47.1 ± 10.6) and the Expanded Disability Status Scale (EDSS; Kurtzke, 1983) to quantify the level of physical and cognitive disability in MS (scores range: 1–10; normal neurological exam-death; mean ± SD = 7.1 ± 2.8). When enrolled in the study, all patients were already using an AT device/solution based on their needs [see Individually Prioritized Problem Assessment (IPPA) and AT in Table 1]. All patients with MS were naïve to BCI protocol.

**TABLE 1:** Information about the neuropsychological assessment, the problems, the assistive technology, and the participation in the h-BCI evaluation for each patient included in the study.

MS patient	Neuropsychological assessment	Problems (IPPA)	Assistive Technology	h-BCI
P1	Executive Functions: **✓** Attention: **✓** Working Memory:✓	Computer accessibility	Mouse emulator: Joystick	✓
P2	Executive Functions: **✗** Attention: **✓** Working Memory:✗	Reading due to fatigue	Mainstream solutions with customized accessibility settings	✓
P3	Executive Functions: **✓** Attention: **✓** Working Memory:✓	Computer and smartphone accessibility	Mouse emulator: Head-tracker	✓
P4	Executive Functions: **✓** Attention: **✓** Working Memory:✓	Computer accessibility	Mainstream solutions with customized accessibility settings	✓
P5	-	Computer accessibility	Mainstream solutions with customized accessibility settings	✓
P6	Executive Functions: **✓** Attention: **✓** Working Memory:✓	-	Mainstream solutions with customized accessibility settings	✓
P7	Executive Functions: **✗** Attention: **✗** Working Memory:✗	Computer accessibility	Mainstream solutions with customized accessibility settings	✓
P8	Executive Functions: **✗** Attention: **✗** Working Memory: ✗	Face to face communication Reading/writing Making phone calls	Customized Grid 3 interface operated with a button switch and a scanning modality	✓
P9	Executive Functions: **✗** Attention: **✗** Working Memory: ✗	Smartphone accessibility Reading	Mainstream solutions with customized accessibility settings	✗
P10	Executive Functions: **✓** Attention: **✓** Working Memory:✓	Face to face communication Writing/reading Computer and smartphone accessibility Social interactions Independence in daily activity	Mainstream solutions with customized accessibility settings	✗
P11	Executive Functions: **✓** Attention: **✗** Working Memory:✓	Computer accessibility Writing/reading	Mouse emulator: Joystick	✗
P12	Executive Functions: **✓** Attention: **✓** Working Memory:✗	Face to face communication Smartphone accessibility Accessibility to entertainment applications.	Customized Grid 3 interface to support access to PC applications, operated with a button switch and a scanning modality	✗
P13	-	Computer and smartphone accessibility Accessibility to domotic system	Head tracker to support access to PC	✗

*Neuropsychological assessment: the column reports the results of clinical neuropsychological assessment for executive functions, attention, and working memory. The mark “**✗**” stands for a deficit, the mark “**✓**” stands for normal cognitive functioning, and “-” stands for the absence of a neuropsychological assessment. Problems (IPPA): the column reports the problems identified by participants by filling in the IPPA (Individually Prioritized Problem Assessment interview; Wessels et al., 2002). IPPA is a semi-structured interview that aims at investigating seven (or fewer) problems (related to communication and environmental interaction in this case) that the patients would like to solve with the AT. Assistive Technology: the column reports the AT solution used by each participant to overcome the problems identified with the IPPA. H-BCI: the column reports the participation (or not) in the h-BCI system evaluation session. The mark “**✗**” is used when the patient completed all the sessions included in the protocol, and the mark “**✓**” is used when the patients did not complete all the tasks included in the protocol.*

### Hybrid Brain-Computer Interface System

We implemented an h-BCI system prototype based on the communication between the AT software Grid 3 (Smartbox Assistive Technology, 2021) and the BCI software BCI2000 (Schalk et al., 2004). The system combines the P300 ERP with conventional input devices (e.g., head tracker and mouse) as input channels, thus resulting in a hybrid control of the system (Grid 3).

**FIGURE 1:**
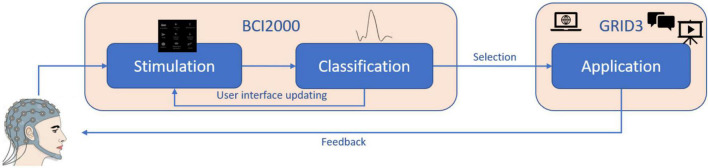
Illustration of the system design and the relationship between the software modules.

Grid 3 is a highly versatile AT software used to create customized interfaces, providing aided access to PC applications, and allowing to combine different input devices (e.g., head tracker and switch). It is one of the most used commercial AT software for communication in AT services. Grid 3 accepts inputs from conventional keyboards and allows to associate a maximum of eight keys to specific and customizable actions to control applications (e.g., F1 can act as “back to home page,” F2 as “jump to keyboard grid,” and F3 as “turn up the volume”). Taking advantage of this feature, we modified the BCI2000 source code (Schalk et al., 2004) so that selections made with the P300-speller application generated a keypress event and activated specific commands associated with the key by means of Grid 3. By doing so, Grid 3 operated as a “link” between BCI2000 and the specific applications (Figure 1; e.g., WhatsApp, YouTube, and Google Chrome).

**FIGURE 2:**
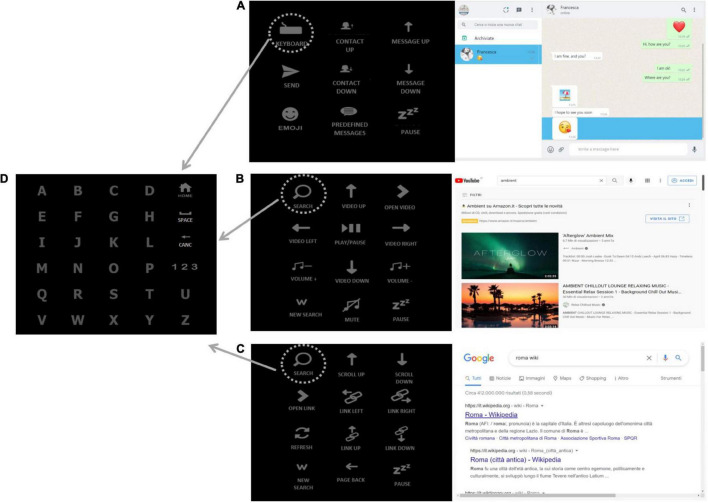
The h-BCI system interfaces. **(A)** *WhatsApp interface:* “contact up” and “contact down” icons allow the user to scroll through the contacts, the “keyboard icon” (in the upper-left corner of the matrix) allows to open the keyboard **(D)** and spell the message, the message is sent by selecting the “send” icon. “Message up” and “message down” icons allow the user to scroll through the messages received within a contact chat. **(B)** *YouTube interface*: the “search” icon (in the upper-left corner of the matrix) is selected to search the desired item by opening the keyboard **(D)** and spelling a keyword, the “arrow” icons (video up, video down, video left, and video right) are selected to scroll through the videos displayed on the screen; “open video” icon allows to choose the desired item and the “play/pause” one to start and stop the video. **(C)** *Google Chrome interface*: the “search” icon (in the upper-left corner of the matrix) is selected to search the desired link, the “scroll up” and “scroll down” icons allow to scroll the links displayed on the screen, “open link” icon allows to choose the link to be opened. **(D)** *Keyboard:* the keyboard matrix is accessed by selecting the “keyboard” **(A)** and the “search” **(B,C)** icons on the main matrices.

To carry out the experimental protocol, we used a 34-inch screen (3,440 × 1,440 pixel, 79.7 × 33.3 cm). The distance between the screen and the patient’s eyes was 100 cm. Grid 3 interfaces were characterized by the presence of a control matrix on the left side of the screen and the application interface on the right side of the screen, where commands were delivered. The control matrix for each application consisted of the main starting matrix (Figures 2A–C) and of a keyboard matrix (6 × 5; Figure 2D), which could be accessed by selecting a specific icon on the main matrix (Figure 2). The interface delivered auditory feedback when a selection was made on control matrices.

WhatsApp control matrix (3 × 3; Figure 2A) allowed to move through contacts and messages and to write and send messages, emojis, and predefined messages. Emojis and predefined messages were organized in two different submatrices (4 × 3); in the h-BCI system control, the latter matrices were accessible by means of a conventional input device (hybrid control).

YouTube control matrix (4 × 3; Figure 2B) allowed to search videos, go through a list of videos, play a video, and control the volume.

Google Chrome control matrix (4 × 3; Figure 2C) allowed to make personalized surfing on the web, scan and select links on a web page, go back to a previous page, and refresh a web page. Each matrix included a command to pause the BCI control.

### Evaluation Protocol

The protocol consisted of two sessions performed on 2 different days.

Session I – *BCI control ability test*: Patients with MS were asked to control a P300-speller (Farwell and Donchin, 1988) to familiarize themselves with a BCI system and test their ability to control a validated BCI system whose reliability has been largely demonstrated (Rezeika et al., 2018). The P300-speller session lasted about 90 min.

Session II – *Grid 3 access *via* AT input and *via* BCI control*: Participants were asked to use Grid 3 to access three applications (WhatsApp, YouTube, and Web Browser) with (1) a conventional commercial AT input device (Grid 3 access *via* a conventional AT input) and (2) with the h-BCI system (Grid 3 access *via* BCI control). Interfaces and tasks were comparable in the two conditions. Data obtained in the two conditions were compared in terms of usability. This session lasted about 120 min.

Sessions I and II were performed within 10 days.

#### Session I: Brain-Computer Interface Control Ability Test

Patients with MS had to operate a P300-speller (Farwell and Donchin, 1988) on a 15′′ screen. The distance between the screen and the patient’s eyes was 60 cm. Scalp EEG signals were acquired using a 16-channel amplifier (g.USBamp, g.tec, Austria; 256 Hz) from 16 sintered Ag/AgCl active electrodes (g.Ladybird, g.tec, Austria) placed according to the 10-10 International System (Fz, Cz, Pz, Oz, P3, P4, PO7, PO8, F3, F4, FCz, C3, C4, CP3, CPz, CP4, referenced to the right ear lobe and grounded to the left mastoid). A conductive gel was applied between the electrodes and the scalp to lower impedances. The impedance value did not exceed 10 kΩ. The P300-speller consisted of a 6 × 5 matrix, which contained 30 alphabetic characters intensified in pseudo-random groups according to the checkerboard stimulation paradigm (Townsend et al., 2010). The stimulus duration was 125 ms and the interstimulus interval (ISI) was 125 ms [stimulus onset interval (SOA): 250 ms].

Participants had to spell eight predefined five-character words (eight runs) with a pause of about 3 min between them. Each target was intensified 16 times, corresponding to eight stimulation sequences, and participants were instructed to attend the target stimulus and mentally count how many times it was intensified. Target characters were cued at the beginning of each trial. No feedback was provided during the first three runs (15 selections; calibration). This EEG data set was used to extract the BCI classifier parameters by applying a Stepwise Linear Discriminant Analysis (SWLDA; Krusienski et al., 2006). We used the BCI classifier parameters to determine the online feedback on the spelling of the following five words (25 selections; online copy mode).

#### Session II: Grid 3 Access *via* Assistive Technology Input and *via* Brain-Computer Interface Control

##### Grid 3 Access *via* Assistive Technology Input

The “Grid 3 access *via* AT input” condition was aimed at evaluating patients’ ability to operate Grid 3 independently from the BCI channel. Patients with MS operated Grid 3 with their own conventional (PC mouse) or alternative input device (e.g., head tracker and switch; see Table 1).

In this condition, the number of selections needed to complete a task varied as a function of the input channel: e.g., the selection of an item operated with a switch and the scanning modality required a double-action (selection of the row and then of the column containing it) in comparison with the selection performed with the mouse emulators, allowing direct control of the cursor (e.g., head tracker and joystick).

Patients with MS were first instructed about the experimental procedures. They were then asked to perform the following tasks:

• Task 1: WhatsApp (selections required: 8 or 16 in case of scanning modality). Participants had to select the second contact in the chat list by scrolling it down, open the keyboard, and write the message “OK,” then they had to go back to the “main menu,” send the message, open the emoji menu, and send the second emoji.
• Task 2: YouTube (selections required: 5 or 10 in case of scanning modality). Participants were required to scroll down the video list and open the second video, turn up the volume, and pause the video.
• Task 3: Google Chrome (selections required: 5 or 10 in case of scanning modality). Participants had to open the second link of a web page, scroll down the page, and select the command “pause.”

##### Grid 3 Access *via* Brain-Computer Interface Control

Patients with MS had to operate Grid 3 using the h-BCI, combining the P300-based BCI and the conventional/alternative input device used in the Grid 3 access *via* AT input condition (hybrid control). Scalp EEG signals were acquired using a 16-channel amplifier (g.USBamp, g.tec, Austria; 256 Hz) from eight sintered Ag/AgCl active electrodes (g.Ladybird, g.tec, Austria) placed according to the 10-10 International System (Fz, Cz, Pz, Oz, P3, P4, PO7, PO8, referenced to the right ear lobe and grounded to the left mastoid). A conductive gel was applied between the electrodes and the scalp to lower impedances. Each h-BCI system session consisted of a “Calibration” and an “Online mode.” Patients with MS were instructed to focus their attention on the target and mentally count how many times it was intensified. The experimenter pointed at the target stimuli.

Calibration consisted of six runs (4 items each; 24 total items) with matrices of different sizes: two runs with a 3 × 3 matrix, two runs with a 4 × 3 matrix, and two runs with a 6 × 5 matrix. No feedback was provided to participants. Items were randomly intensified by rows and columns for 125 ms and with an ISI of 125 ms; each item was intensified 30 times (15 stimulation sequences). The EEG data set collected during the calibration was used to extract the BCI classifier parameters by applying an SWLDA.

The “online mode” consisted of three runs; patients were asked to perform the same tasks performed during the “Grid 3 access *via* AT input” condition. The number of stimulation sequences was optimized for each participant. The criterion applied to establish the number of sequences to be used in the online mode was *n* + 1, where “*n*” was the number of sequences necessary to reach 100% offline accuracy (applying a sixfold cross-validation procedure to the data recorded in the calibration). Feedback occurred at the end of each trial.

Task 1: WhatsApp (minimum number of selections required: 8 or 9 in case of hybrid control based on scanning modality). Only the emoji (one out of eight selections) was selected with the hybrid control (conventional/alternative input device); the remaining seven were BCI-based selections. This leads to variability in the number of selections.

Task 2: YouTube (minimum number of selections required: 5).

Task 3: Google Chrome (minimum number of selections required: 5).

In case of wrong selection, the participant had to correct the error.

The tasks were interrupted if the participants reached the k × 3 number of selections (where “*k*” is the minimum number of selections expected to complete the task).

### Outcome Measures

#### Session I: Brain-Computer Interface Control Ability Test

P300-speller performance was evaluated as follows and then compared with h-BCI system performance:

• Online accuracy (%) is defined as the ratio between correct selections and selections needed to complete the task.
• The highest written symbol rate (WSR; Furdea et al., 2009) was assessed as a function of the number of stimulus repetitions delivered in a given trial of the five online copy-mode runs. The maximum WSR value for each subject provides an objective evaluation of the system performance by combining the accuracy level with the time needed to reach it, in terms of the number of stimulation sequences. In the “copy-mode” runs, the participants were asked to (copy) spell predefined words. In case of errors, the participants were not asked to correct the wrong selection; he/she had to proceed with the next letter of the word. Therefore, the number of selections was fixed.

#### Session II: Grid 3 Access *via* Assistive Technology Input and *via* Brain-Computer Interface Control

The usability of the system in the two conditions of session II (Grid 3 access *via* AT input and Grid 3 access *via* BCI control) was evaluated in terms of effectiveness, efficiency, and satisfaction (ISO, 2019).

• *Effectiveness* is defined as the accuracy and completeness with which the user achieves goals while using the system. It was evaluated as follows:
  • Online accuracy (%): Calculated by dividing the number of correct selections by the total number of selections.
  • Completeness: Indicated with the number of participants who completed the protocol. The tasks were not considered complete after the n × 3 number of selections (with “n” as the minimum number of selections needed to complete the task).
• *Efficiency* describes the degree to which the system enables quick, effective, and economic performance in terms of time, human effort, costs, and materials. It was evaluated in terms of workload.
  The workload was assessed through the National Aeronautics and Space Administration – Task Load Index (NASA-tlx; Hart, 2006). NASA-tlx is a multidimensional questionnaire that assesses perceived workload during the usage of a high technology device. It was administered by an experimenter at the end of both the “Grid 3 access *via* AT input” and “Grid 3 access *via* BCI control” conditions. The overall workload score (0–100) is a weighted average between the rating of six factors (i.e., mental demand, physical demand, temporal demand, performance, effort, and frustration level). Each factor has a weighted rating that ranges from 0 to 33.33. Higher scores are associated with higher levels of workload.
• *Effectiveness/efficiency*: Time per correct selection and WSR were considered metrics belonging to both effectiveness and efficiency constructs:
  • WSR (Furdea et al., 2009) was assessed offline as a function of the number of stimulus repetitions delivered in a given trial of calibration; it was considered the highest offline WSR value. WSR was computed only for the “Grid 3 access *via* BCI control.”
  • Time for correct selection (s) was computed as the ratio between the total time to complete the online tasks and the number of correct selections.
• *Satisfaction* represents the degree to which the user’s physical, cognitive, and emotional responses that result from the use of a system meet the user’s needs and expectations. It was evaluated by means of the System Usability Scale (SUS; Bangor et al., 2009). SUS is a 5-point Likert scale that assesses user satisfaction with a technological device. Participants were required to express their agreement/disagreement with the statements on the scale. It was administered by an experimenter at the end of both conditions.

### Statistical Analysis

We investigated the correlation between the P300-speller accuracy and the h-BCI system accuracy by means of Spearman’s rank test. These analyses aimed at demonstrating the reliability of the h-BCI, assuming the reliability of the well-validated P300-speller.

#### Usability Assessment

To evaluate whether the introduction of the P300-based BCI as an additional channel to control the AT software would affect system usability, we compared the “Grid 3 access *via* AT input” condition with the “Grid 3 access *via* BCI control” condition for the five patients who successfully controlled the h-BCI. We compared the two conditions in terms of effectiveness (accuracy), efficiency (time per correct selection, NASA-tlx scores), and satisfaction (SUS scores) scores by means of a (non-parametric) Wilcoxon matched-pairs test since the distributions violated the assumption of normality. Regarding the NASA-tlx, we compared both the overall perceived workload score and the single-factor scores (mental demand, physical demand, temporal demand, performance, effort, frustration) by means of a (non-parametric) Wilcoxon matched-pairs test. Finally, any possible correlations between the accuracy in “Grid 3 access *via* BCI control” condition and FSS scores (level of fatigue) and EDSS scores (level of disability) were investigated by means of Spearman’s rank test to evaluate whether the level of fatigue and disability could influence the ability to control the h-BCI.

## Results

### Pilot Evaluation

We first evaluated the h-BCI system, including a convenience sample of 13 healthy volunteers (mean age = 27.2 ± 2.9; nine women), with no history of neurological/psychiatric disorders. All participants were able to control the system with a mean (±SD) online accuracy of 98.1 ± 2.7% and a mean (±SD) time per correct selection 25.3 s (±8.1). The mean (± SD) overall perceived workload (NASA-tlx) was 39.1 (±18.2), and the mean (± SD) satisfaction score (SUS) was 81.4 (± 12.6).

### Session I: Brain-Computer Interface Control Ability Test

A total of 11 of 13 patients with MS participated in session I (two women; mean age: 52 ± 14.0; mean time since diagnosis: 248.8 ± 133.2 months; years of formal education: 13 ± 3.1 years). Three patients gained a control accuracy of the P300-speller below the 50% (Table 2; P5, P7, P8) and showed a WSR = 0, not supporting an efficient communication. Two of those patients had an accuracy of 0%. A total of 8 of 11 patients had a WSR > 2, showing an efficient control of the P300-speller.

**TABLE 2:** Performance data in sessions I and II.

MS patient	Session I BCI control ability test (P300-speller)			Session II			
				Grid 3 access *via* AT input		Grid 3 access *via* BCI control	
	Acc (%)	WSR	Task completion	Acc (%)	Task completion	Acc (%)	Task completion
P1	56	2.7	**✓**	100	**✓**	79.0	**✓**
P2	88	2.4	**✓**	87.8	**✓**	62.8	**✓**
P3	96	9.1	**✓**	100	**✓**	80.4	**✓**
P4	100	9.4	**✓**	100	**✓**	94.4	**✓**
P5	44	0	**✗**	100	**✓**	46.5	**✗**
P6	92	6	**✓**	93.3	**✓**	100	**✓**
P7	0	0	**✗**	85.4	**✓**	11.4	**✗**
P8	0	0	**✗**	87.6	**✓**	40	**✗**
Mean ± SD	59.5 ± 41.7			94.3 ± 6.5		64.3 ± 30.2	
P9	96	3.4	**✓**	100%	**✓**	–	–
P10	96	6.1	**✓**	–	–	–	–
P13	96	4.9	**✓**	–	–	–	–
Mean ± SD	69.4						

*Acc, accuracy; WSR, written symbol rate. Task completion: the mark “**✓**” stands for the complete task, the mark “**✗”** stands for the incomplete task, and the mark “–” means that the patient did not participate in that task.*

### Session II: Grid 3 Access *via* Assistive Technology Input

A total of 9 of 13 patients with MS participated in the “Grid 3 access *via* AT input” condition (two women; mean age: 54.9 ± 10.6; mean ± SD time since diagnosis: 249.2 ± 128.1 months; mean education: 13 ± 3.5 years).

• *Effectiveness*: All patients with MS completed the tasks (Tables 2, 3), with a mean accuracy of 94.9 ± 6.4%.

**TABLE 3:** The mean (± SD) group results, including the patients who completed each session.

Outcome measures		Session I BCI control ability test	Session II	
			Grid 3 access *via* AT input	Grid 3 access *via* BCI control
	Patients (n)	8/11	9/9	5/8
Effectiveness	Online accuracy (%)	90 ± 14.2	94.9 ± 6.4	83.3 ± 14.6
Efficiency	NASA-tlx (Total workload; 0–100)	–	22.3 ± 20.8	24.4 ± 21.0
Effectiveness/Efficiency	WSR (sym/min)	5.5 ± 2.7	–	4.4 ± 3.7
	Time for correct selections (s)	–	8.8 ± 6.3	41.0 ± 16.2
Satisfaction	SUS (0–100)	–	78.1 ± 10.8	78 ± 16.6

*Session I, patients with WSR > 0; Session II, patients who completed the three tasks.*

• *Efficiency*: Mean time per correct selection of the nine participants in the session was 8.8 s (±6.3). Total workload (NASA-tlx) was 22.3 (±20.8) with weighted ratings of NASA-tlx factors ranging from 1.2 (effort) to 8.1 (mental demand) (Table 4).

**TABLE 4:** Scores for the NASA-tlx and SUS questionnaires in session II: Grid 3 access *via* AT input and Grid 3 access *via* BCI control.

Patient	NASA-TLX							SUS
	Workload tot	Mental demand	Physical demand	Temporal demand	Performance	Effort	Frustration	
**Grid 3 access *via* AT input**								
P1	4	1.3	1.3	1.3	0	0	0	95
P2	60.3	31.7	6.7	10	10	2	0	70
P3	6.7	0	2.7	1.3	0	2.7	0	87.5
P4	12.6	3.3	1.3	5.3	2	0.7	0	87.5
P5	8.3	2.7	0.3	2.7	1	1.7	0	62.5
P6	0	0	0	0	0	0	0	82.5
P7	30.3	10	0	0	2.7	2.7	15	67.5
P8	39.3	16	4.7	4.7	4	0	10	72.5
Mean ± SD	20.2 ± 21.1	8.1 ± 11	2.1 ± 2.4	3.2 ± 3.4	2.5 ± 3.4	1.2 ± 1.2	3.1 ± 5.9	78.1 ± 11.6
**Grid 3 access *via* BCI control**								
P1	4.7	1.3	1	1.7	0	0.7	0	90
P2	46.7	23.3	5	1.3	1.3	15	0.7	57.5
P3	40.3	10.7	4	1.3	4.7	4.7	25	62.5
P4	30.3	18.3	0	2.7	0	9.3	0	92.5
P5	48.3	18.7	2.7	24	0	1.3	1.7	72.5
P6	0	0	0	0	0	0	0	87.5
P7	29.7	13.3	0.3	3.3	6.7	2.7	3.3	75
P8	29.3	8	0	4	8	2.7	6.7	60
Mean ± SD	28.7 ± 17.9	11.7 ± 8.4	1.6 ± 2	4.8 ± 7.9	2.6 ± 3.4	4.5 ± 5.1	4.7 ± 8.5	74.7 ± 14

• *Satisfaction*: SUS score was on average 78.1 (±10.8) (Table 4).

### Session II: Grid 3 Access *via* Brain-Computer Interface Control

Eight patients with MS participated in the “Grid 3 access *via* BCI control” condition (two women; mean age: 54.1 ± 11.1; mean time since diagnosis: 249.5 ± 137.0 months; mean education: 13 ± 3.8 years).

• *Effectiveness*. Five out of eight patients with MS (P1, P2, P3, P4, and P6) controlled the h-BCI system since they completed the three online tasks. Three participants (P5, P7, and P8) did not complete the online tasks (Tables 2, 3). Patients with MS that controlled the h-BCI system obtained a mean online accuracy of 83.3% ± 14.6% (Table 3).
• *Efficiency*. The five patients with MS that controlled the system obtained a mean (± SD) time per correct selection of 41.0 s ± (16.2) and a mean offline WSR of 4.4 sym/min (±3.7); the mean (±SD) overall workload score was 24.4 (±21.0) in patients with MS that controlled the h-BCI; weighted ratings of NASA-tlx factors ranged from 1.2 (performance) to 10.7 (mental demand). The mean (±SD) overall workload score was 35.8 (±10.9) in patients with MS that did not control the h-BCI system, and the weighted ratings of NASA-tlx factors ranged from 1 (physical demand) to 13.3 (mental demand) (Table 4).
• *Satisfaction*. The mean (±SD) SUS score (0–100) was 78 (±16.6) in the five patients with MS that were able to control the h-BCI system and 69.2 (±8.0) in the three patients with MS who did not control the h-BCI system (Table 4).

### Brain-Computer Interface Control Ability Test vs. Grid 3 Access *via* Brain-Computer Interface Control

Considering all the eight patients with MS who participated in session II, we found a significant correlation between the P300-speller online copy-mode accuracy and the h-BCI system accuracy (Grid 3 access *via* BCI control condition; r_*s*_ = 0.9, *p* < 0.05).

The three patients with MS (P5, P7, P8) who did not complete the three tasks in the “Grid 3 access *via* BCI control” condition also did not control the P300-speller (accuracy < 50%, WSR = 0).

### Usability Assessment

#### Effectiveness

**FIGURE 3:**
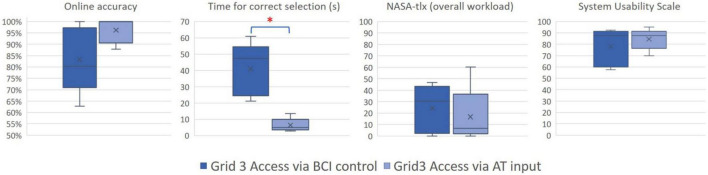
Box plots compare the results for the two control mode conditions: Grid 3 Access *via* BCI control (left) and Grid 3 access *via* AT input (right). *Online accuracy*: the ratio between correct selections and selections needed to complete the task; *time for correct selection*: the ratio between the total time to complete the tasks and the number of correct selections; *NASA-tlx:* the overall workload scores in the NASA-tlx questionnaire*; System Usability Scale:* level of satisfaction evaluated with the SUS. Box plots represent the distribution of the measurements, whiskers reach from minimum to maximum, the lines depict the medians, the “x” depicts the mean level, and the boxes cover the values between the first and third quartile. * indicates a significant difference (*p* < 0.05).

All the eight patients who participated in both the “Grid 3 access *via* AT input” condition and the “Grid 3 access *via* BCI control” condition successfully completed the “Grid 3 access *via* AT input” condition. Five of them successfully completed the “Grid 3 access *via* BCI control” condition (Table 2). Considering the five patients with MS who completed both the conditions, we did not find significant differences in the accuracy (*Z* = 1.5; *p* = 0.1; Figure 3).

No significant correlations were found between the h-BCI system online accuracy and the FSS scores (r_*s*_ = 0.29, *p* = 0.49) and the EDSS scores (r_*s*_ = –0.57, *p* = 0.013), respectively.

#### Efficiency

Considering the five patients with MS who completed both the “Grid 3 access *via* AT input” condition and the “Grid 3 access *via* BCI control” condition, we did not find significant differences in workload scores (NASA-tlx scores: overall workload: *Z* = 1.1, *p* = 0.3; mental demand: *Z* = 1.1, *p* = 0.3; physical demand: *Z* = 0.9, *p* = 0.4; temporal demand: *Z* = 1.1, *p* = 0.3; performance: *Z* = 0.5, *p* = 0.6; effort: *Z* = 1.8, *p* = 0.1; frustration: *Z* = 1.3, *p* = 0.2). We found a significant difference in time per correct selection, which was significantly higher in the “Grid 3 access *via* BCI control” condition (*Z* = 2.0, *p* < 0.05; Figure 3).

#### Satisfaction

Considering the five participants who successfully controlled the h-BCI system, no significant differences were found between the SUS scores in the “Grid 3 access *via* AT input” condition and in the “Grid 3 access *via* BCI control” condition (*Z* = 0.9, *p* = 0.3; Figure 3).

## Discussion

We implemented an h-BCI system combining the P300-based BCI technology with commercial AT input devices to access a range of computer applications through a widely used AT software for communication and environmental interaction: Grid 3. We evaluated the usability of the h-BCI system involving 13 patients with MS who were admitted to the AT service of Fondazione Santa Lucia, IRCCS (Rome, Italy). Patients participated in two sessions, including (i) the control of the P300-speller (BCI control ability test; Farwell and Donchin, 1988) and (ii) the access to WhatsApp, YouTube, and Web Browser through Grid 3 first with a conventional commercial input device and then with the h-BCI system (Grid 3 access *via* AT input and *via* BCI control).

**FIGURE 4:**
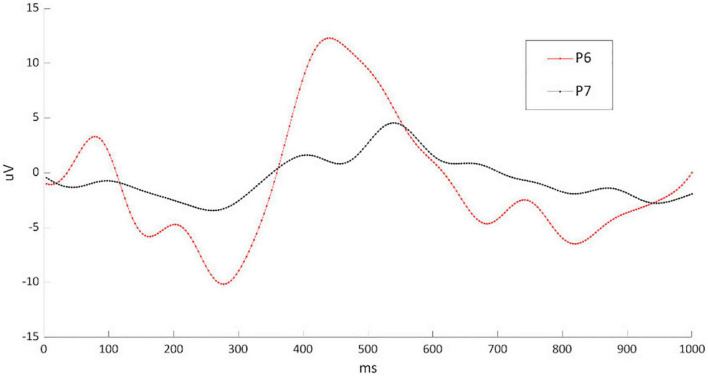
Illustration of the P300 waveform in Pz from the participant who best controlled the h-BCI system (P6; in red) and the participant who worst controlled the system (P7; in black). EEG data sets were high-pass and low-pass filtered with cutoff frequencies of 0.1 and 10 Hz, respectively; a notch filter was used to remove 50 Hz contamination. EEG signal was segmented in epochs of 1 s starting at the onset of each stimulus. A baseline correction was done based on the average EEG activity within 200 ms immediately preceding each epoch. The average waveform for both target and non-target epochs was computed for each trial to assess P300 peak amplitude and latency. The ERP waveforms were obtained from a sample-by-sample contrast between the target and non-target ERP waveform amplitude and therefore show the difference between target and non-target; the analysis was conducted on the data sets from the six calibration runs.

First, we tested the reliability of the newly developed h-BCI system referring to the stand-alone P300-speller, assuming the P300-speller as the most validated BCI. The comparison showed that those patients who successfully controlled the P300-speller also succeeded in mastering the h-BCI system. On the other hand, the three patients who were not able to control the h-BCI system showed similar “illiteracy” for the P300-speller control (Table 2; P5, P7, P8). Overall, these data underlined the reliability of the h-BCI system and allow us to infer that the inability to control the h-BCI system was due to patients’ peculiarities (Oreja-Guevara et al., 2019; see below) rather than the system features. As shown by way of example in Figure 4, the amplitude of the P300 waveform over Pz for the participant who best controlled the h-BCI system (P6) was, at a visual inspection, higher with respect to the participant who did not control the system (P7). Furthermore, we noted that patients who did not control the h-BCI system showed a deficit in the executive functions, attention, and working memory (as for P5 the neuropsychological assessment was not available; Table 1). We, therefore, hypothesize that cognitive impairments could, in part, account for the inability to control the h-BCI system. This was consistent with previous studies that found a significant involvement of cognitive abilities in BCI performance in patients with amyotrophic lateral sclerosis (Riccio et al., 2013, 2018; Geronimo et al., 2016) and underlines the importance to consider the cognitive abilities in the implementation of BCI-based AT devices.

Patients with MS have been rarely considered as potential BCI end-users. To the best of our knowledge, only one previous study (Martinez-Cagigal et al., 2016) reported the evaluation of a P300-based BCI (for the web browser access) with 16 patients with MS. The authors reported a control accuracy of 84.14(±10.08)% which is comparable with our results (83.3% ± 14.6% accuracy), with three patients who had a classification accuracy in the calibration session of <70%, and were then excluded from the assessment.

In addition to the investigation of the ability to control the system in patients with MS, we evaluated the h-BCI system usability according to the UCD metrics (Kübler et al., 2014; Riccio et al., 2015; Schettini et al., 2015). The comparison between the two conditions (Grid 3 access *via* AT input and BCI control) did not reveal significant differences both in terms of effectiveness and users’ satisfaction. As for the efficiency, the access to Grid 3 was faster when conventional input devices were used compared with the BCI input. However, this disadvantage (in terms of time resources involved by the user) in the efficiency of the h-BCI system was not confirmed by the workload as perceived by patients with MS (NASA-tlx), which was comparable between the two conditions. Also, participants’ satisfaction was not affected in the h-BCI system despite the h-BCI system time demand. Thus, we can infer that taking into account the (well-known) high time demand of the BCI channel, the BCI addition as a control channel of the AT software does not worsen the usability of the whole system. However, we must consider that these results could be influenced by an effect of the fascination with high technologies associated with the fact that patients performed brief tasks proposed only once. Further developments aimed at addressing the time demand issue and consequently at improving the system efficiency would include the asynchronous control (dynamic stopping of the stimulation) and automatic control suspension features together with the automatic recalibration of the classifier’s parameters (Schettini et al., 2014; Guy et al., 2018). Also, such developments would concern the evaluation of classifiers potentially more accurate than the SWLDA and the evaluation of their feasibility with the proposed system; e.g., BLDA classifier, (non)linear SVM (Manyakov et al., 2011), and Riemannian classifiers (Delgado et al., 2020). Moreover, the fact that our results did not show any relation between the system control and the patients’ level of fatigue underlines the importance of deeply investigating such an issue to hypothesize an advantage of the h-BCI system to access ATs.

Although cautious due to the limited sample size and the fact that the study was conducted in an experimental setting, these preliminary findings support the reliability of a P300-based BCI as an additional input channel to access a commercial AT and the evidence that such an additional channel has no additional costs on users’ perception of usability with respect to muscular-based aids. Future studies involving a larger cohort of patients should be performed to improve the power of the statistical analysis and to better investigate the potential of this (hybrid) approach in real-life scenarios. This would allow considering more variables (e.g., the comfort of the EEG cap montage, including electrodes characteristics, the use of the conductive gel, the essential presence of a skilled operator), and their influence on patients’ perceived satisfaction. Furthermore, to overcome the possibility that a unique evaluation session would lead patients to overestimate the usability of the system, a longitudinal study in an ecological setting would be the next step.

Finally, we consider the integration of the BCI with the daily/commercial AT devices and the involvement of AT services in the development of innovative devices and in their customization and validation as an important step for the BCI inclusion in AT services portfolio of solutions.

## Data Availability Statement

The raw data supporting the conclusions of this article will be made available by the authors, without undue reservation.

## Ethics Statement

The studies involving human participants were reviewed and approved by the Local Ethical Committee of Fondazione Santa Lucia, IRCCS. The patients/participants provided their written informed consent to participate in this study.

## Author Contributions

AR was responsible for the experimental design, data collection, analysis of data, and manuscript writing. FS was responsible for the experimental design, h-BCI system implementation, analysis of data, and manuscript writing. VG contributed to participants’ enrollment, data collection and analysis, and manuscript writing. EG contributed to participants’ enrollment and data collection. MG contributed to participants’ enrollment. DM and FC supervised the overall experimental design implementation, data interpretation, and manuscript editing. All authors contributed to the article and approved the submitted version.

## Conflict of Interest

The authors declare that the research was conducted in the absence of any commercial or financial relationships that could be construed as a potential conflict of interest.

## Publisher’s Note

All claims expressed in this article are solely those of the authors and do not necessarily represent those of their affiliated organizations, or those of the publisher, the editors and the reviewers. Any product that may be evaluated in this article, or claim that may be made by its manufacturer, is not guaranteed or endorsed by the publisher.
